# Tamoxifen Inhibition of Kv7.2/Kv7.3 Channels

**DOI:** 10.1371/journal.pone.0076085

**Published:** 2013-09-24

**Authors:** Tania Ferrer, Ivan Arael Aréchiga-Figueroa, Mark S. Shapiro, Martin Tristani-Firouzi, José A. Sanchez-Chapula

**Affiliations:** 1 Unidad de Investigación “Carlos Méndez” del Centro Universitario de Investigaciones Biomédicas de la Universidad de Colima, Colima, Col. México; 2 Department of Physiology, University of Texas Health Science Center, San Antonio, Texas, United States of America; 3 Nora Eccles Harrison Cardiovascular Research and Training Institute, University of Utah, Salt Lake City, Utah, United States of America; 4 Division of Pediatric Cardiology, University of Utah, Salt Lake City, Utah, United States of America; Sackler Medical School, Tel Aviv University, Israel

## Abstract

KCNQ genes encode five Kv7 K^+^ channel subunits (Kv7.1–Kv7.5). Four of these (Kv7.2–Kv7.5) are expressed in the nervous system. Kv7.2 and Kv7.3 are the principal molecular components of the slow voltage-gated M-channel, which regulates neuronal excitability. In this study, we demonstrate that tamoxifen, an estrogen receptor antagonist used in the treatment of breast cancer, inhibits Kv7.2/Kv7.3 currents heterologously expressed in human embryonic kidney HEK-293 cells. Current inhibition by tamoxifen was voltage independent but concentration-dependent. The IC_50_ for current inhibition was 1.68 ± 0.44 µM. The voltage-dependent activation of the channel was not modified. Tamoxifen inhibited Kv7.2 homomeric channels with a higher potency (IC_50_ = 0.74 ± 0.16 µM). The mutation Kv7.2 R463E increases phosphatidylinositol- 4,5-bisphosphate (PIP_2_) - channel interaction and diminished dramatically the inhibitory effect of tamoxifen compared with that for wild type Kv7.2. Conversely, the mutation Kv7.2 R463Q, which decreases PIP_2_ -channel interaction, increased tamoxifen potency. Similar results were obtained on the heteromeric Kv7.2 R463Q/Kv7.3 and Kv7.2 R463E/Kv7.3 channels, compared to Kv7.2/Kv7.3 WT. Overexpression of type 2A PI(4)P5-kinase (PIP5K 2A) significantly reduced tamoxifen inhibition of Kv7.2/Kv7.3 and Kv7.2 R463Q channels. Our results suggest that tamoxifen inhibited Kv7.2/Kv7.3 channels by interfering with PIP_2_-channel interaction because of its documented interaction with PIP_2_ and the similar effect of tamoxifen on various PIP2 sensitive channels.

## Introduction

*KCNQ* genes encode members of the voltage-gated delayed rectifier K^+^ channels, Kv7 family. There are five members of this family, from Kv7.1 to Kv7.5 and only four of these Kv7.2-Kv7.5 are confined to the nervous system, Kv7.1 is restricted to the heart and peripheral epithelial and smooth muscle cells [1]. Heterotetrameric Kv7.2/Kv7.3 channels are believed to underlie the neuronal M current, a noninactivating, slowly deactivating, subthreshold current [2], the M current stabilizes the membrane potential in the presence of depolarizing currents and contributes to the resting potential of neurons [3]. Genetic deficiency or acute inhibition of M channels in neurons leads to overexcitability (eg, seizures), whereas M channel openers have an antiexcitatory effect [4].

M-channels are inhibited by a variety of neurotransmitters and hormones acting on G protein-coupled receptors, principally those coupling to Gq and/or G11. Muscarinic-1 (M1) receptor agonists activate Gq to stimulate phospholipase C_β_ and catalyse the hydrolysis of membrane phosphatidylinositol- 4,5-bisphosphate (PIP_2_) [5]. The reaction produces the two classical second messengers, soluble inositol 1,4,5- trisphosphate and membrane-delimited diacylglycerol. PIP_2_ is localized to the cytoplasmic leaflet of the plasma membrane where it regulates ion channel and transporter activity. Direct regulation of Kv channels by PIP_2_ has been reported for many Kv channels including the Kv7 family [6-9]. Kv7 (and native M) channels require PIP_2_ in order to enter the open state and close when membrane PIP_2_ levels are reduced or its polar head groups neutralized [7,8,10-12]. Channel closure occurs as a result of a reduction in membrane PIP_2_ levels.

Inhibition of the M current by muscarinic agonists or by drugs such as amitriptyline or linopirdine, initiates seizure activity in vitro and in vivo [13,14]. The M1-agonist oxotremorine-M inhibits M current in rat superior cervical ganglion (SCG) neurons by reducing PIP_2_ levels. The inhibition of the current was assayed in vitro to test the effect of oxotremorine-M in Kv7.2 mutants with different PIP_2_ affinities. Channel inhibition by oxotremorine-M was stronger for channels that weakly interact with (PIP_2_) [15].

Tamoxifen is a nonsteroidal mixed antiestrogenic agent that is a competitive antagonist at the estrogen receptor [16,17] and has been used to treat breast cancer in postmenopausal women since 1971 [18]. Tamoxifen acts also as a multichannel blocker that inhibits several potassium conductances in cardiac tissue, including I_K1_, I_to_, I_sus_, and I_Kr_ [19-22]. Recently, we found that clinically relevant concentrations of tamoxifen reduce heterologously expressed Kir2.x inward rectifier potassium channel current and IK1 in native cardiac myocytes [23]. Kir2.x channels, like the Kv7 family, require PIP_2_ for function [24]. We demonstrated that the inhibitory effects of tamoxifen on Kir2.x channels were due to interference with the interaction of the channels and membrane (PIP_2_) [23]. Tamoxifen also inhibits cardiac ATP-sensitive and acetylcholine-activated K+ currents in part by interfering with PIP_2_ [25]. Here, we tested the hypothesis that tamoxifen inhibits Kv7.2/Kv7.3 channels via a PIP_2_ mechanism, similar to that reported for Kir2.x.

## Materials and Methods

### DNA constructs and transfection of HEK-293 cells

Wild-type (WT) cDNAs encoding both human Kv7.2 and rat Kv7.3 cloned in pcDNA3.1 (+) plasmid (Invitrogen, CA, USA) and the Kv7.2 mutants R463E and R463Q subcloned in pGW1-CMV (British Biotechnology, Oxford, United Kingdom) and PIP5K 2A cloned in pBluescript (Agilent Technology, CA, USA) were transiently transfected with Lipofectamine 2000 reagent (Invitrogen, Carlsbad, CA) according to the manufacturer’s instructions into human embryonic kidney (HEK-293) cells as previously described [26]. The cDNA encoding type 2A PI(4)P5-kinase (PIP5K 2A) was kindly provided by Dr. Giscard Seebohm (Ruhr-University, Bochum, Germany). For electrophysiological experiments, cells were used 24 h after transfection. As a marker for successfully transfected cells, cDNA encoding green fluorescent protein (GFP) was cotransfected together with the cDNAs of the genes of interest.

### Electrophysiological recordings

Ionic currents were measured using the whole-cell configuration of the standard patch-clamp technique [27] at room temperature. Signals were amplified using an Axopatch 200B patch-clamp amplifier (Molecular Devices, California, USA) and filtered at 1 kHz. Patch electrodes were pulled with a Flaming/Brown micropipette puller (Sutter Instruments, California, USA) and fire-polished to a final resistance of 2-6 MΩ when filled with internal solution. Data acquisition was achieved using pClamp 8.1 software (Molecular Devices, California, USA).

The external recording solution contained (in mM) 130 NaCl, 4 KCl, 1.8 CaCl_2_, 1 MgCl_2_, 10 HEPES, and 10 glucose, pH 7.4. The pipette solution contained (in mM) 110 KCl, 10 HEPES, 5 K _4_BAPTA, 5 K _2_ATP, and 1 MgCl_2_, pH 7.2. KCNQ currents were determined as the current that was sensitive to block by 50 µM XE-991.

To generate current-voltage (I-V) relationships, pulses were applied in 10-mV increments at a frequency of 0.05 Hz. Test potentials ranged from -80 to +40 mV and were applied for 1.5 s from a holding potential of -80 mV. Deactivating (tail) currents were measured at -60 mV. Currents measured at the end of 1.5 s test pulses were used to measure steady state fractional block (“fraction blocked”), defined as the amplitude of current reduced by drug divided by control current amplitude.

### Drugs

Tamoxifen (Tocris Bioscience, USA) was dissolved in ethanol as a 100 mM stock solution and diluted in the external solution just before use, at the required concentration. HEK-293 cells were exposed to drug solution until steady-state effects were achieved, usually in about 10 minutes. To determine the concentration-effect relationships, a single cell was exposed to only one concentration of the drug. XE-991(Tocris Bioscience, Bristol, United Kindom) was dissolved in distilled deionized water as a stock solution and then diluted in the external solution at 50 µM.

### Data analysis

Recordings were analyzed using Clampfit 9.0 (Molecular Devices) and Origin 7 (OriginLab Corp., Northampton, MA) software. Quantitative data are presented as mean ± S.E.M. (*n* corresponds to the number of cells), asterisks denote significance. Currents were normalized to the current recorded at +40 mV under control conditions. Concentration-response relationship was constructed plotting the fractional block of the current (*f*) at +40 mV versus the drug concentration ([D]). The data were fitted with a Hill equation, *f* = 1/{1+(IC_50_)/[D]^*n*^_H_}, to determine the concentration (IC_50_) required for 50% inhibition of current magnitude and the Hill coefficient, *n*_H_.

The voltage dependence of Kv7.2/Kv7.3 activation was determined from tail currents measured at -60 mV following 1.5-s test depolarizations. Normalized tail current amplitude (In) was plotted versus test potential (V_t_) and fitted to a Boltzmann function, In=1/(1+ exp[(V_1/2_−V_t_) / k ]), using Origin software (Northampton, MA). V_1/2_ is the voltage at which the current is half-activated, and k is the slope factor of the relationship.

The time constants for deactivation (τ_deactivation_) were determined by fitting current decay with a monoexponential function using Clampfit 9.0 (Molecular Devices).

Statistical analyses were performed by using the Statistica (Tulsa, OK) software package, version 10.0. Concentration-response data were tested for significance by Student’s *t* test applying Bonferroni’s. A two-tailed Student’s *t* test was applied to compare individual data sets (*t* test; 5% significance level).

## Results

### Tamoxifen inhibits Kv7.2/Kv7.3 channels

**Figure 1:**
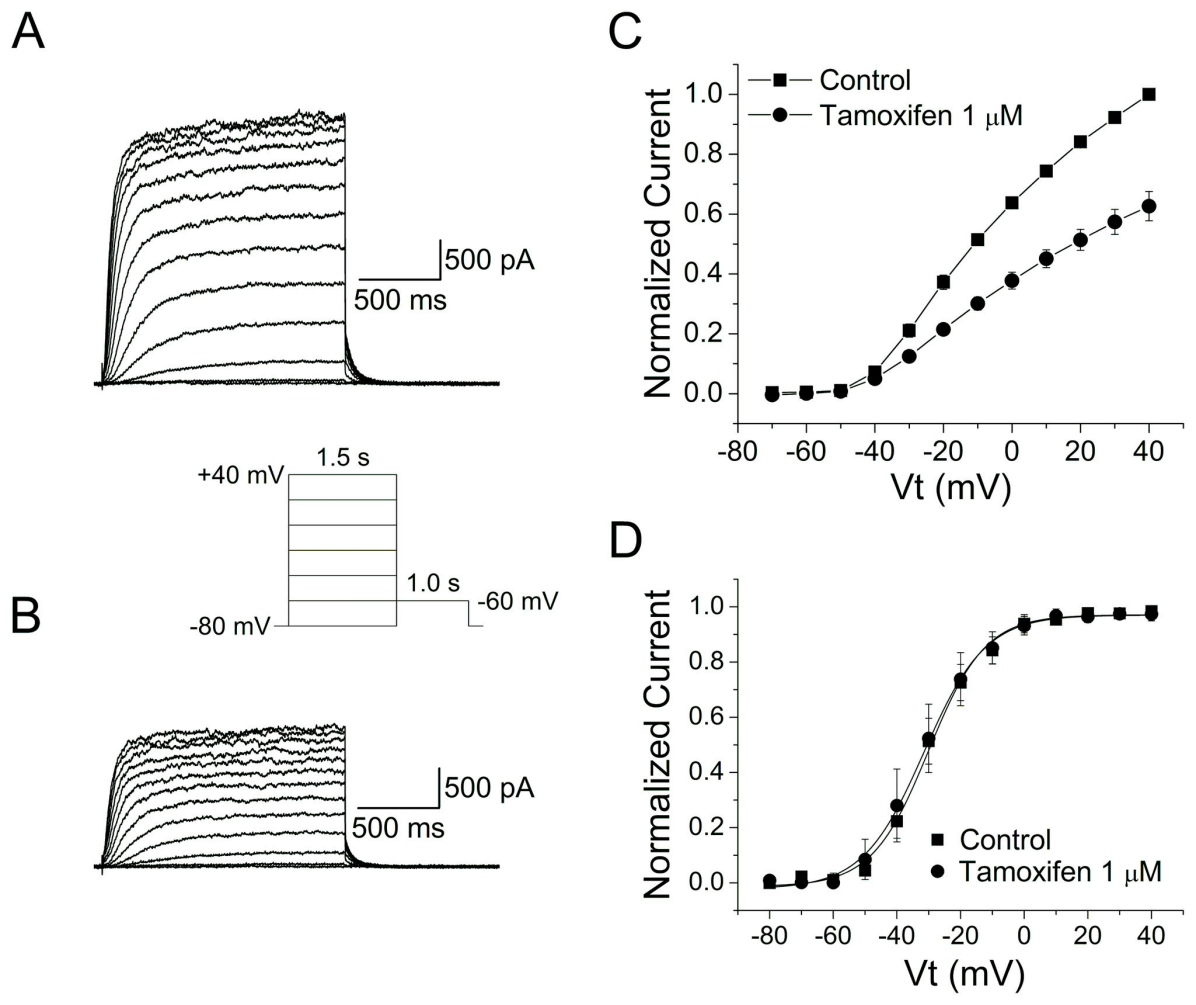
Tamoxifen inhibited Kv7.2/Kv7.3 channels expressed in HEK-293 cells. A and B, ionic currents recorded in control condition (A) and in presence of 1 µM tamoxifen (B) using voltage protocol shown in the inset. C, Current-voltage relationship in control (square, n = 6) and in presence of 1 µM tamoxifen (circles, n = 6). D, Kv7.2/Kv7.3 activation curves in control (squares, n = 5) and in presence of 1 µM tamoxifen (circles, n = 6).

The effects of tamoxifen on Kv7.2/Kv7.3 channels were investigated in the whole-cell configuration in transfected HEK-293 cells. Currents were elicited by 1.5-s depolarizing pulses to potentials ranging from -80 to +40 mV followed by repolarization to -60 mV. The voltage protocol was repeated every 15 s. Each drug concentration was perfused for 10 min to obtain a steady state effect. An example of Kv7.2/Kv7.3 currents recorded before and after inhibition by 1 µM tamoxifen is shown in Figure 1, A and B. The inhibitory effect of tamoxifen on Kv7.2/7.3 channels for currents measured at the end of a 1.5-s test pulse was quantified as the reduction of the maximum outward current at a membrane potential of +40 mV. The normalized I-V relationship (Figure 1C) shows that 1 µM tamoxifen inhibit Kv7.2/Kv7.3 channels by 40% at +40 mV (n = 6). This effect was irreversible. Current inhibition by tamoxifen was not due to a shift in the activation curve. The amplitude of tail current as a function of test potential was fitted with a Boltzmann function; this relationship had a V_1/2_ of -30.07 ± 0.72 mV and a slope factor of 8.89 ± 0.64 (n = 6) in control conditions. In the presence of tamoxifen 1 µM, the V_1/2_ was not significantly shifted (V_1/2_ was -31.48 ± 0.57 with a k of 9.6 ± 0.51, n = 6) (Figure 1D).

**Figure 2:**
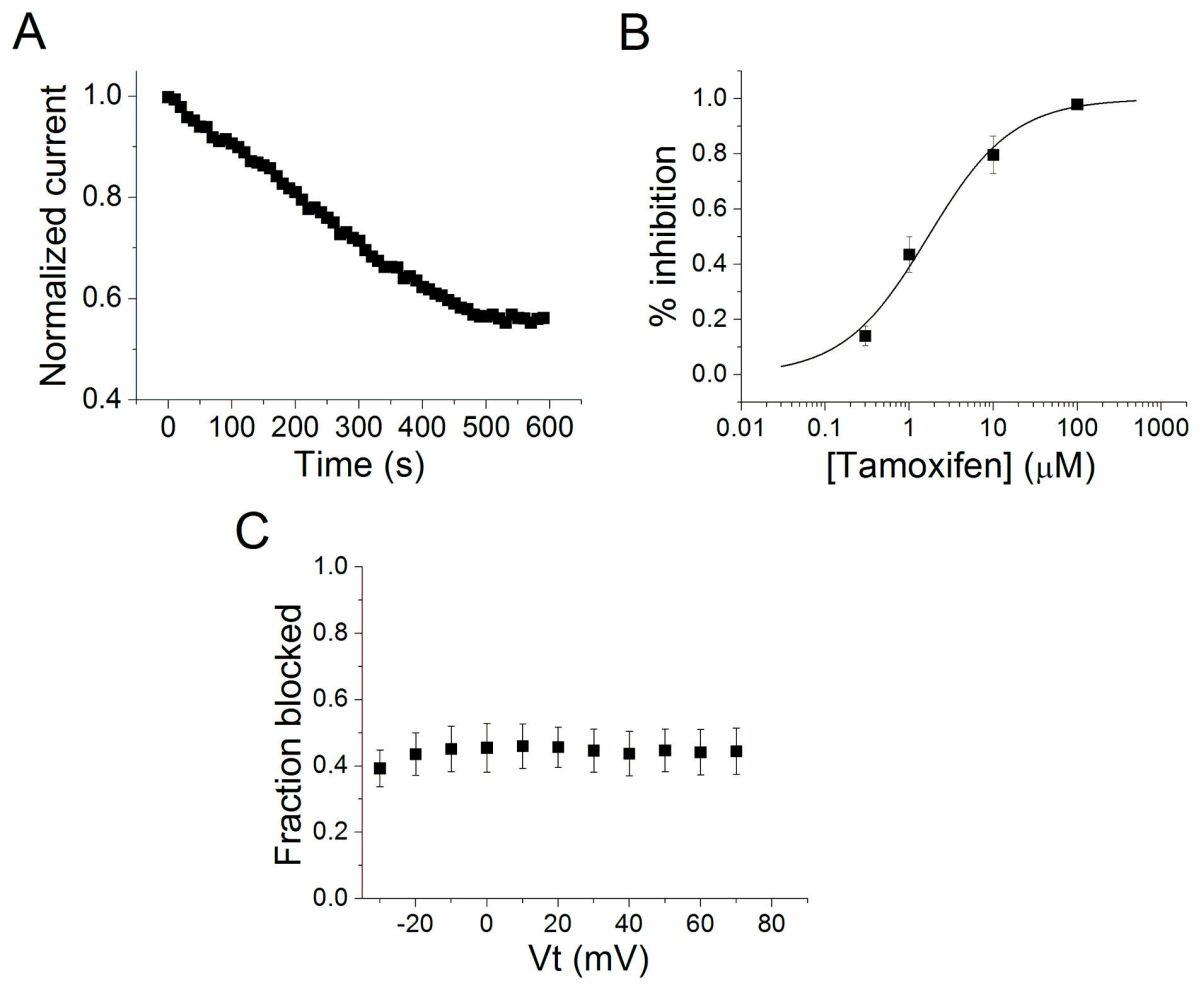
Kv7.2/Kv7.3 current inhibition by tamoxifen is voltage independent but concentration-dependent. A, Time course of the inhibitory effect of 1 µM tamoxifen on Kv7.2/Kv7.3 current (n = 6). B, Concentration–effect relationship for block of Kv7.2/Kv7.3 channels by tamoxifen. IC_50_ = 1.68 ± 0.44 µM, n_H_ = 0.86 ± 0.12(n = 6). The line represents the fit of the experimental data by a Hill equation with the values given in the text. C, Fractional block of Kv7.2/Kv7.3 current produced by 1 µM of tamoxifen as voltage function (n=5).

The time course of tamoxifen inhibition of Kv7.2/7.3 currents measured at + 40 mV is shown in Figure 2A. The time course of 1 µM tamoxifen inhibition was slow with a half-maximal inhibitory time of 220 ± 17 s (n = 6). The effect of tamoxifen on Kv7.2/Kv7.3 channels was concentration-dependent but voltage-independent. The concentration–response curve of the effects of tamoxifen on Kv7.2/Kv7.3 channels is shown in Figure 2B. IC_50_ value for current inhibition was 1.68 ± 0.44 µM and the Hill coefficient was 0.86 ± 0.12 (n = 6). A plot of the maximum current amplitudes measured at the end of 1.5 s pulses indicates that inhibition of Kv7.2/Kv7.3 channels by tamoxifen (1 µM) was voltage-independent (Figure 2C).

### Homomeric Kv7.2 channels are inhibited by tamoxifen and tamoxifen inhibition correlates with the apparent affinity for PIP_2_

While Kv7.2 and Kv7.3 channels form heteromeric channels underlying the M-current [2], each channel alone can also form functional homomeric channels [2,28,29]. As homomeric Kv7.2 and heteromeric Kv7.2/Kv7.3 channels have distinct PIP_2_ affinities [7], we tested whether Kv7.2 and Kv7.2/Kv7.3 channels also differ in their sensitivity to tamoxifen. Kv7.3 channels express poorly in heterologous systems [30] and thus the effect of tamoxifen on Kv7.3 homomeric channels was not assayed.

**Figure 3:**
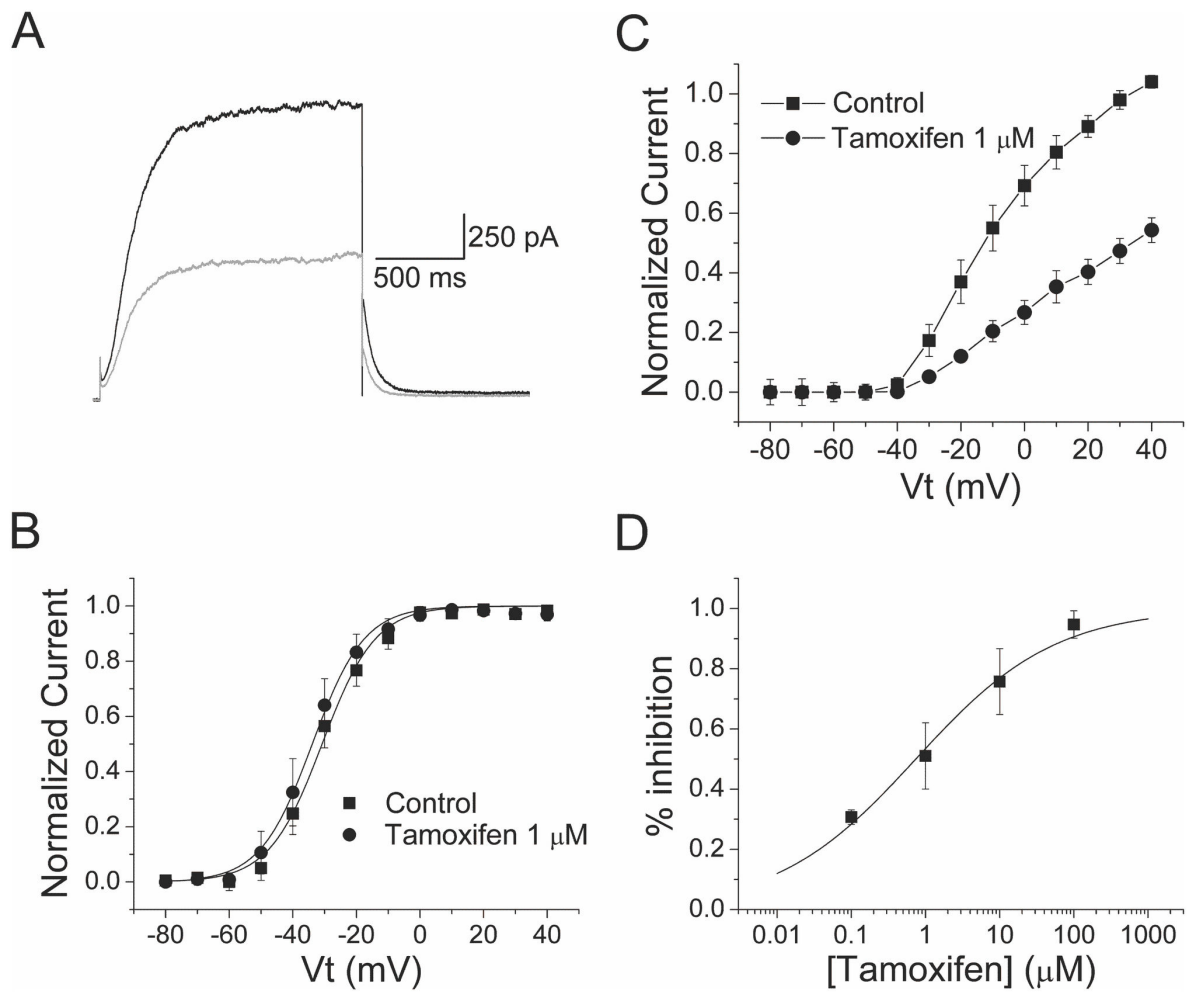
Homomeric Kv7.2 channels are inhibited by tamoxifen. A, Current traces of Kv7.2 in presence and absence of tamoxifen 1 µM. B, Current-voltage relationship in control (squares, n = 5) and in presence of 1µM tamoxifen (circles, n = 5). C, Kv7.2 activation curves in control (squares, n = 5) and in presence of 1 µM tamoxifen (circles, n = 5). D, Concentration–effect relationship for block of Kv7.2 channels by tamoxifen. IC_50_ = 0.74 ± 0.16 µM, n_H_ = 0.4 ± 0.05(n = 5). The line represents the fit of the experimental data by a Hill equation with the values given in the text.

The inhibitory characteristics of tamoxifen on Kv7.2 homomeric channels are shown in Figure 3. Tamoxifen (1 µM) inhibited about 54% of the current at +40 mV (Figure 3). Current inhibition was not related to a shift in the activation curve of the channel. The V_1/2_ and a slope factor was -23.15 ± 0.54 mV and 8.45 ± 0.48 (n = 5) for Kv7.2 in control condition and -19.77 ± 0.53 mV and 9.06 ± 0.47 (n = 5) in the presence of tamoxifen 1 µM respectively (n = 5, Figure 3C). The effect of tamoxifen on Kv7.2 channels was also voltage-independent (data not shown).

Tamoxifen inhibited Kv7.2 homomeric channels with a higher potency compared to heteromeric Kv7.2/Kv7.3. IC_50_ for tamoxifen on Kv7.2 current was 0.74 ± 0.16 µM (n=5, Figure 3E), which is approximately 2.3 times smaller than that obtained for Kv7.2/Kv7.3 channels (p<0.05).

**Figure 4:**
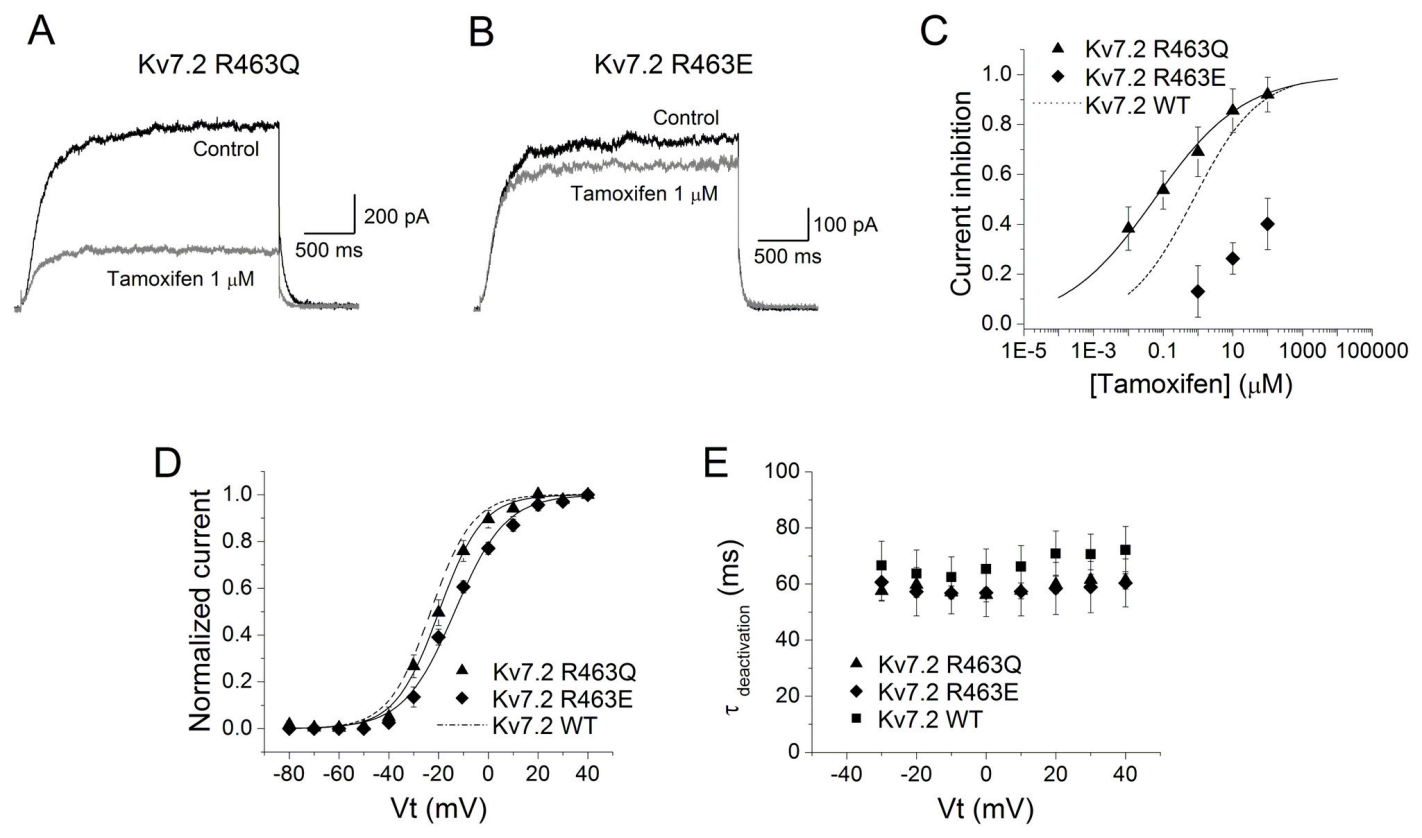
Tamoxifen inhibition of Kv7.2 channels is related to PIP_2_-channel affinity. A and B, Superimposed representative Kv7.2 R463Q and Kv7.2 R463E current traces recorded in control (black) and in presence of 10 µM tamoxifen (gray). Currents were evoked depolarizing the membrane to +40 mV for 3 s and then repolarizing to -60 mV. C, Concentration–effect relationship for the inhibitory effect of tamoxifen on Kv7.2 WT channels (dashed line) and the mutants Kv7.2 R463Q (triangles) and Kv7.2 R463E (diamonds). The line represents the fit of the experimental data by a Hill equation with the values given in the text. Each point represents the mean ± SEM from *n* = 5 to 6 experiments. D, Activation curves for Kv7.2 WT (dashed line, n = 5), Kv7.2 R463Q (triangles, n = 6) and Kv7.2 R463E (diamonds, n = 6). E, Deactivation time constants resulting from the monoexponential fit of the tail current decay (τ_deact_) at -60 mV after a test pulse to +40 mV, are shown for Kv7.2 WT (n = 5), Kv7.2 R463Q (n = 6) and Kv7.2 R463E (n = 6).

To further explore the relationship between the channel affinity for PIP_2_ and the degree of tamoxifen inhibition, we selected Kv7.2 mutant channels with reduced PIP_2_ affinity (R463Q) and increased affinity (R463E) [31]. Figure 4 (A and B) shows current traces measured at -60 mV after a 3 s pulse to +40 mV for R463Q, and R463E Kv7.2 channels in control condition and in presence of 1 µM tamoxifen. Tamoxifen induced weak inhibitory effects on the mutant Kv7.2 R463E channels; the highest concentration tested (100 µM) produced ~40% current inhibition (Figure 4C). Because of the poor water solubility of tamoxifen, we could not test concentrations higher than 100 µM. Conversely, tamoxifen inhibited Kv7.2 R463Q channels with at least 1000 fold higher potency (IC_50_ of 0.06 ± 0.01 µM, n = 6). Thus, the R463Q mutation increased tamoxifen potency by a factor of twelve compared to Kv7.2 WT channels while R463E caused more than fifty-fold reduction in drug potency. These data support the idea that the degree of tamoxifen inhibition is related to the channel’s intrinsic sensitivity to PIP_2_; that is, tamoxifen inhibition was greater for channels that weakly interacted with PIP_2_.

In order to exclude the possibility that the differential tamoxifen inhibition was a result of preferential stabilization or binding to the closed channel state, we analyzed the voltage dependence of activation for Kv7.2 R463Q and Kv7.2 R463E (Figure 4D). The mutation R463Q (which decreases open probability as a consequence of reduced PIP_2_-channel interaction [31]) did not significantly change the activation voltage dependence of the channel (V_1/2_ = -19.85 ± 0.46 mV, slope 8.73 ± 0.41 mV, n = 6) compared to Kv7.2 WT (V_1/2_ = -23.15 ± 0.54 mV and slope 8.45 ± 0.48). The time constant for deactivation was not altered by the R463Q mutation (τ_deactivation_ = 61.3 ± 3.1 ms, n = 6, R463Q vs 66.1 ± 8.4 ms, n = 5, WT Kv7.2). However, the potency of tamoxifen to inhibit Kv7.2 R463Q was twelve fold higher than WT Kv7.2. The Kv7.2 R463E mutant (which increased open probability as a consequence of increased PIP_2_-channel interaction [31]) shifted significantly the voltage dependence of activation to the right (V_1/2_ = -13.53 ± 0.43, n = 6), compared to Kv7.2 WT (V_1/2_ = -23.15 ± 0.54, n = 5), but did not alter the slope factor (10.23 ± 0.62 for Kv7.2 R463E vs 8.45 ± 0.48 for Kv7.2 WT). However, the R463E mutation did not alter the rate of channel deactivation (τ_deactivation_ = 60.36 ± 8.56 ms, n = 6). Taken together, these results suggest that tamoxifen inhibition is most likely related to the channel’s intrinsic sensitivity to PIP_2_ and not related to preferential binding or stabilization of the closed channel state.

**Figure 5:**
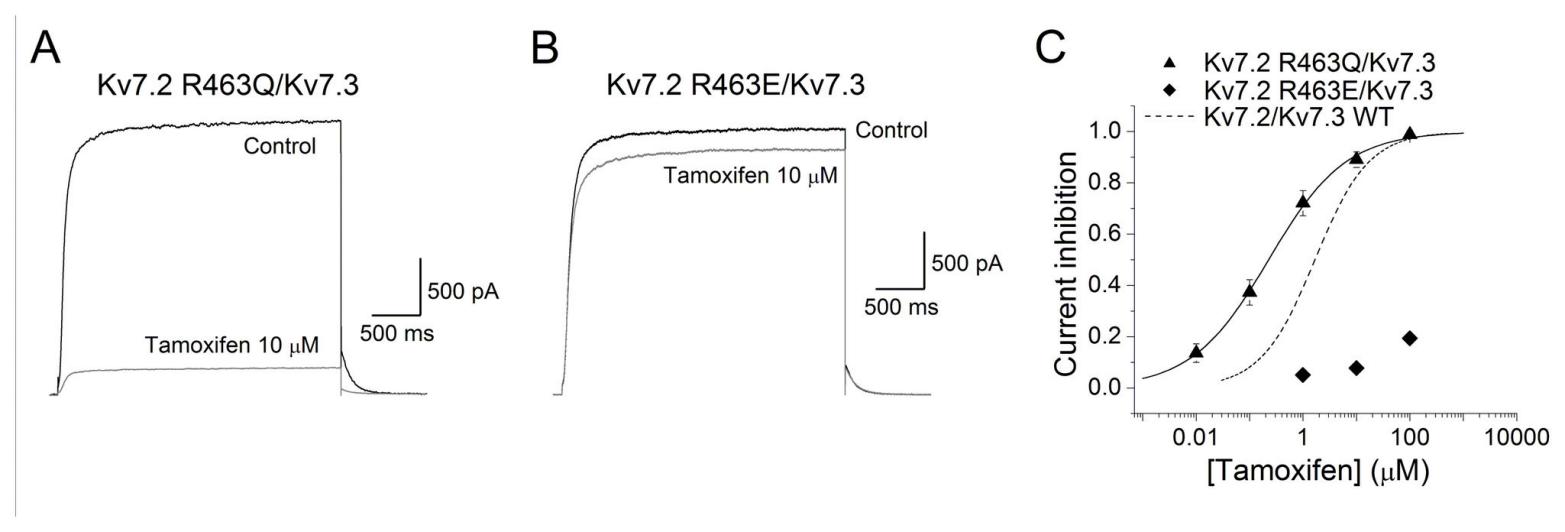
The affinity of the heteromeric mutant channels Kv7.2 R463Q/Kv7.3 and Kv7.2 R463E/Kv7.3 for PIP_2_ correlates with the degree of current inhibition by tamoxifen. A and B, Superimposed representative Kv7.2 R463Q/Kv7.3 and Kv7.2 R463E/Kv7.3 current traces recorded in control (black) and in presence of 10 µM tamoxifen (gray). Currents were evoked depolarizing the membrane to +40 mV for 3 s and then repolarizing to -60 mV. C, Concentration–effect relationship for the inhibitory effect of tamoxifen on Kv7.2/Kv7.3 WT channels (dashed line) and the mutants Kv7.2 R463Q/Kv7.3 and Kv7.2 R463E/Kv7.3 (triangles and diamonds respectively). The line represents the fit of the experimental data by a Hill equation with the values given in the text. Each point represents the mean ± SEM from *n* = 5 to 6 experiments.

In addition, as homomeric Kv7.2 and heteromeric Kv7.2/Kv7.3 channels have distinct PIP_2_ affinities [7], and tamoxifen affects Kv7.2 and Kv7.2/Kv7.3 differentially, we studied the effect of the drug on the Kv7.2 R463Q and Kv7.2 R463E, in a heteromeric conformation with Kv7.3 subunits. Figure 5 (A and B) shows current traces when the cells were depolarized to +40 mV then hyperpolarized to -60 mV to induce the deactivation tail current for Kv7.2 R463Q/Kv7.3, and Kv7.2 R463E/Kv7.3 channels in control condition and in presence of 10 µM tamoxifen. Tamoxifen exerted weaker inhibitory effects on the mutant Kv7.2 R463E/Kv7.3 channels; the highest concentration tested (100 µM) produced ~ 20% current inhibition (Figure 5C). Conversely, tamoxifen inhibited Kv7.2 R463Q/Kv7.3 channels with a higher potency (IC_50_ of 0.22 ± 0.01 µM, n = 6). Thus, tamoxifen potency to inhibit the heteromeric mutant channels Kv7.2 R463Q/Kv7.3 was increased by a factor of eight compared to Kv7.2/Kv7.3 WT channels while the potency of the drug to inhibit Kv7.2 R463E/Kv7.3 was decreased more than seventy-fold.

### Inhibition of Kv7.2/Kv7.3 and Kv7.2 R463Q currents by tamoxifen is reduced by overexpression of PIP5K-2A

**Figure 6:**
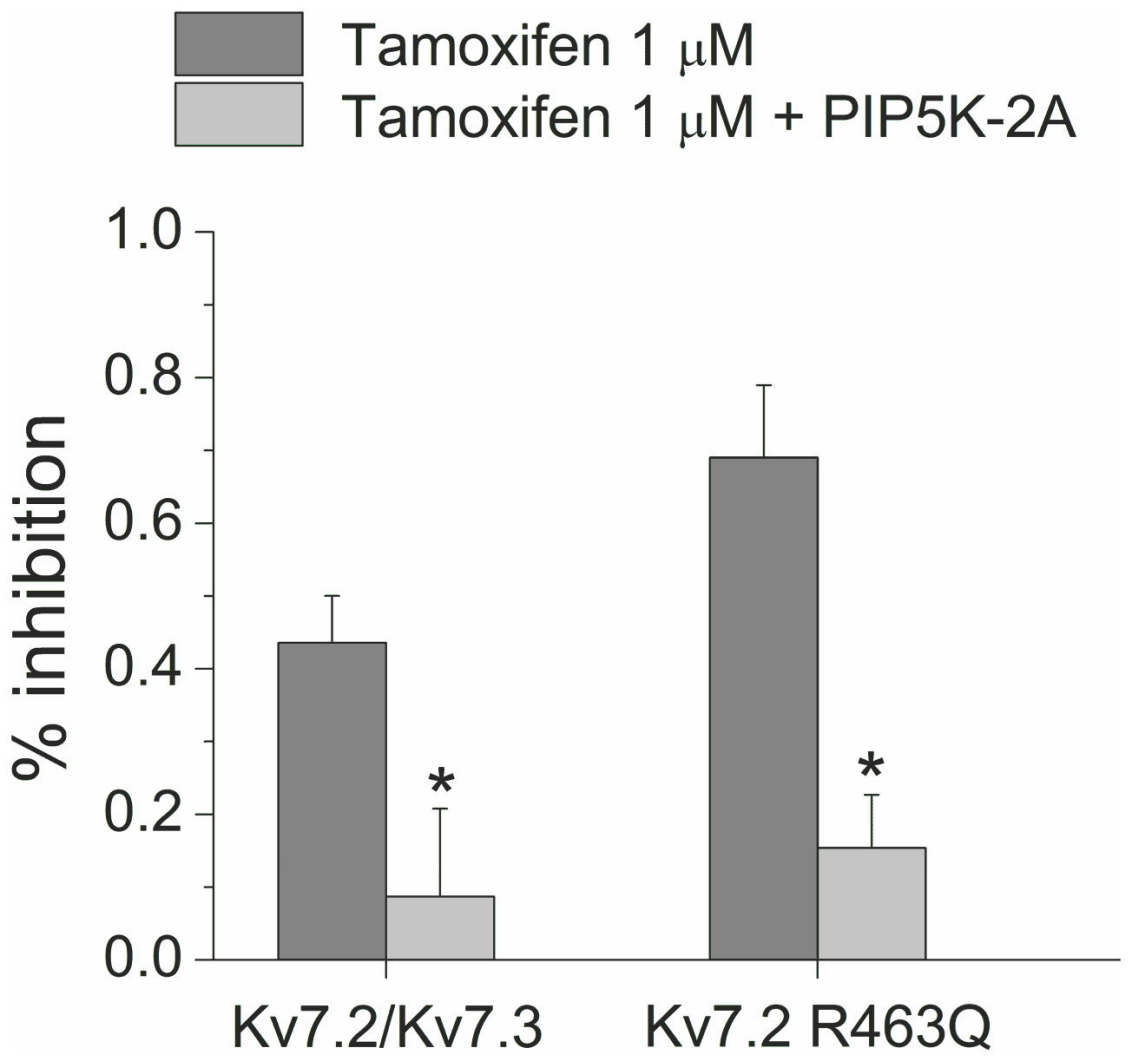
Overexpression of PIP5K-2A diminished the effect of tamoxifen on Kv7.2/Kv7.3 and Kv7.2 R463Q channels. Inhibition of the currents recorded at 40 mV by tamoxifen 1 µM when Kv7.2/Kv7.3 and Kv7.2 R463Q channels were expressed alone compared to the effect of tamoxifen 10 µM when those channels were co-expressed with the kinase.

It was previously reported that muscarinic-induced inhibition of both expressed Kv7.2/7.3 channels and native neuronal M-channels is reduced or prevented when membrane PIP_2_ levels are increased by over-expressing the PIP5-kinase, which synthesize PIP_2_ [32,33]. To further characterize the possible relationship between tamoxifen and PIP_2_ we explored the effect of tamoxifen on Kv7.2/Kv7.3 and Kv7.2 R463Q current after co-expressing those channels and PIP5K 2A. Overexpression of the kinase decreased significantly the inhibitory effect of tamoxifen on Kv7.2/Kv7.3 current (p <0.05). Tamoxifen at a concentration of 10 µM inhibited only 9% of the current (Figure 6, n = 5) in the presence of kinase overexpression, compared to 43% current inhibition in its absence. Likewise, the effect of tamoxifen significantly diminished when the mutant Kv7.2 R463Q was co-expressed with PIP5K 2A (p <0.05). Tamoxifen 10 µM reduced current by 15% in presence of PIP5K 2A (n = 6) versus 70% reduction by 1 µM tamoxifen in the absence of kinase overexpression. These results further underscore the relationship between PIP_2_ activity and tamoxifen inhibition of Kv7.2 homomultimeric mutant and heteromultimeric channels.

## Discussion

In the present study we found that Kv7.2/Kv7.3 channels were inhibited by tamoxifen in an irreversible, voltage-independent and concentration-dependent manner. The voltage-dependent activation of the channel was not modified by tamoxifen. Time course of current inhibition by tamoxifen was slow, resembling the run-down phenomenon of Kv7.2/Kv7.3 channels observed in inside-out/outside-in patches. This run-down phenomenon occurs as consequence of PIP_2_ depletion [7]. Kv7 channels are highly sensitive to the PIP_2_ concentration at the cytoplasmic face of the membrane and different family members display a differential apparent affinity for PIP_2_. PIP_2_ stabilizes the allosteric conformational change that opens the channel, such that Po is increased at all voltages. Our data suggest that tamoxifen inhibition is related to the intrinsic ability of the channel to bind PIP_2_. The affinity of Kv7.2/Kv7.3 for PIP_2_ is 5-fold higher compared than Kv7.2 [7] and likewise, tamoxifen was less potent at inhibiting Kv7.2/Kv7.3 heteromeric channels than the Kv7.2 homomeric channels.

The mutation Kv7.2 R463E, which increases PIP_2_ interaction, diminished dramatically the inhibitory effect of tamoxifen compared with that for wild type Kv7.2. In addition, the substitution of the Arg463 in Kv7.2 by a glutamine decreased PIP_2_-channel interaction, shifting the concentration-effect curve to the left, increasing tamoxifen potency. Moreover, the heteromeric channels Kv7.2 R463Q/Kv7.3 and Kv7.2 R463E/Kv7.3 behave similar to the homomeric channels, the affinity of the channel for PIP_2_ correlates with the degree of current inhibition by tamoxifen.

The inhibition order (from strong to weak) for the channels we studied was Kv7.2 R463Q > Kv7.2 R463Q/Kv7.3 > Kv7.2 > Kv7.2/Kv7.3 > Kv7.2 R463E > Kv7.2 R463E/Kv7.3. The potency of tamoxifen to inhibit Kv7.2 and Kv7.2/Kv7.3 channels depends on whether the channels have a relatively low or high apparent affinity for PIP_2_. In support of this hypothesis we found that tamoxifen inhibition of Kv7.2/Kv7.3 and Kv7.2 R463Q channels was significantly reduced when the channel was cotransfected with PIP5-2A kinase. Overexpression of PIP5-kinase experimentally increased PIP_2_ levels [34] potentiating PIP_2_-channel interactions. These results correlate with the fact that agonist-induced Kv7.2/7.3-current suppression is reduced by overexpression of PIP 5-kinase [15] and suggest that tamoxifen probably could be inserted into the lipid membrane.

The effect of tamoxifen on the mutants Kv7.2 R463Q and Kv7.2 R463E suggest that tamoxifen inhibition is not related to tamoxifen binding to and stabilizing the channel closed state. The shift to the right in the activation voltage dependence of Kv7.2 R463E (which is more resistant to tamoxifen inhibition) compared to Kv7.2 WT could indicate that the potency of tamoxifen to inhibit the mutant is smaller because it could be blocking the closed state of the channel. However, the mutation Kv7.2 R463Q (which is more sensitive to tamoxifen inhibition), did not change significantly the activation voltage dependence of the channel compared to Kv7.2 WT. If tamoxifen was blocking the closed state of the channel, we should expect that potency to inhibit Kv7.2 R463Q and Kv7.2 WT channels were very similar. Conversely, the mutation R463Q increased tamoxifen potency by a factor of twelve compared to Kv7.2 WT.

We recently found that cationic amphiphylic drugs like tamoxifen inhibit Kir channels with slow time course and in a voltage-independent manner [23,25]. Tamoxifen inhibits Kir 2.x channels indirectly by interfering with the interaction between the channel and PIP_2_ [23,25]. All members of inward potassium rectifier channel family require PIP_2_ for functioning, like Kv7 channels [9] and tamoxifen seems to inhibit Kir and Kv7.3/Kv7.2 channels by the same mechanism, interfering with PIP_2_-channel interactions.

Tamoxifen alone can mildly stimulate phosphatidylinositol kinase and phosphatidylinositol-phosphate (PIP) kinase activity in GH4C1 membrane preparation and this effect of tamoxifen was strongly synergized by vanadate [35]. However, Friedman and cols. suggest that the tamoxifen stimulation of phosphoinositide kinase activity could be explained by the binding of tamoxifen to PIP and PIP_2_ [36], thus releasing the kinases from the product inhibition. Tamoxifen stimulates phosphoinositide kinase activity with an ED_50_ of 20 µM, in the presence of vanadate. The IC_50_ value for current inhibition by tamoxifen was 1.68 µM for Kv7.2/7.3 WT and 0.74 µM for Kv7.2 WT. At these concentrations tamoxifen without vanadate practically did not modify PIP_2_ membrane concentration [35], and our experiments were performed in the absence of vanadate.

Tamoxifen is a cationic amphiphilic drug (CAD) and it has been shown that CADs bind to the negatively charged phosphoinositides [36]. The cationic group of CADs is normally placed between the polar head groups of phospholipids, and the hydrophobic portion is directed toward the hydrophobic interior of the membrane; thus, the drug molecule intercalates between lipid molecules [37]. We propose that lipophilic compounds such as tamoxifen may insert in the lipid membrane bilayer and interfere with the interaction between Kv7.2/7.3 channel and PIP_2_.

In summary the results of this study demonstrate that tamoxifen inhibits Kv7.2/7.3 channels in a concentration-dependent, voltage-independent and irreversible manner. Our results suggest that tamoxifen inhibited Kv7.2/7.3 channels interfering with PIP_2_-channel interaction because of its documented interaction with PIP_2_ and the similar effect of tamoxifen on various PIP_2_ sensitive channels.
